# Correction: Sherzad et al. Lesion-Symptom Mapping of Acute Speech Deficits After Left vs. Right Hemisphere Stroke: A Retrospective Analysis of NIHSS Best Language Scores and Clinical Neuroimaging. *Brain Sci.* 2025, *15*, 1329

**DOI:** 10.3390/brainsci16101015

**Published:** 2026-09-24

**Authors:** Nilofar Sherzad, Jeremy Yeaton, Roger Newman-Norlund, John Absher, Gregory Hickok, Leonardo Bonilha, William Matchin, Christopher Rorden, Julius Fridriksson, Sigfus Kristinsson

**Affiliations:** 1 Arnold School of Public Health, University of South Carolina, Columbia, SC 29208, USA; nsherzad@email.sc.edu; 2 Department of Neurological Surgery, University of California—Davis, Davis, CA 95616, USA; jyeaton@uci.edu; 3 Department of Psychology, University of South Carolina, Columbia, SC 29208, USA; rnorlund@mailbox.sc.edu (R.N.-N.); rorden@mailbox.sc.edu (C.R.); 4 School of Medicine, University of South Carolina, Greenville, SC 29605, USA; absher@mailbox.sc.edu; 5 Department of Cognitive Sciences & Language Sciences, University of California—Irvine, Irvine, CA 92697, USA; gshickok@uci.edu; 6 Department of Neurology, University of South Carolina, Columbia, SC 29201, USA; leonardo.bonilha@uscmed.sc.edu; 7 Department of Communication Sciences and Disorders, University of South Carolina, Columbia, SC 29208, USA; matchin@mailbox.sc.edu (W.M.); fridriks@mailbox.sc.edu (J.F.)

**Addition of Authors**

Jeremy Yeaton, William Matchin, and Gregory Hickok were not included as authors in the original publication [1]. They contributed to the conceptualization and the investigation of the study and reviewed and approved the manuscript; their roles are reflected in the corrected Author Contributions statement below. This authorship change was requested and agreed upon by all original and added authors. The authors state that the scientific conclusions are unaffected.

The revised authorship and affiliation listed below:

**Updated Authorship**: Nilofar Sherzad ^1^, Jeremy Yeaton ^2^, Roger Newman-Norlund ^3^, John Absher ^4^, Gregory Hickok ^5^, Leonardo Bonilha ^6^, William Matchin ^7^, Christopher Rorden ^3^, Julius Fridriksson ^7^ and Sigfus Kristinsson ^7,^*

**Updated Affiliation:**

1. Arnold School of Public Health, University of South Carolina, Columbia, SC 29208, USA; nsherzad@email.sc.edu
2. Department of Neurological Surgery, University of California—Davis, Davis, CA 95616, USA; jyeaton@uci.edu
3. Department of Psychology, University of South Carolina, Columbia, SC 29208, USA; rnorlund@mailbox.sc.edu (R.N.-N.); rorden@mailbox.sc.edu (C.R.)
4. School of Medicine, University of South Carolina, Greenville, SC 29605, USA; absher@mailbox.sc.edu
5. Department of Cognitive Sciences & Language Sciences, University of California—Irvine, Irvine, CA 92697, USA; gshickok@uci.edu
6. Department of Neurology, University of South Carolina, Columbia, SC 29201, USA; leonardo.bonilha@uscmed.sc.edu
7. Department of Communication Sciences and Disorders, University of South Carolina, Columbia, SC 29208, USA; matchin@mailbox.sc.edu (W.M.); fridriks@mailbox.sc.edu (J.F.)

* Correspondence: sigfus@mailbox.sc.edu; Tel.: +1-803-553-4689

**Updated Author Contributions:** Conceptualization, J.A., R.N.-N., S.K., J.Y., W.M., and G.H.; methodology, J.A., R.N.-N., S.K., and N.S.; software, L.B., and C.R.; validation, S.K., and N.S.; formal analysis, S.K., and N.S.; investigation, J.A., S.K., J.Y., W.M., and G.H.; resources, J.F., and C.R.; data curation, J.A., and R.N.-N.; writing—original draft preparation, S.K., and N.S.; writing—review and editing, J.A., L.B., J.F., S.K., R.N.-N., C.R., N.S., J.Y., W.M., and G.H.; visualization, S.K.; supervision, S.K.; project administration, J.A., J.F., and S.K.; funding acquisition, S.K. All authors have read and agreed to the published version of the manuscript.

This correction was approved by the Academic Editor. The original publication has also been updated.
