# Supplementary material for: Lesion-Symptom Mapping of Acute Speech Deficits After Left vs. Right Hemisphere Stroke: A Retrospective Analysis of NIHSS Best Language Scores and Clinical Neuroimaging
Source: Brain Sci. 2025 Dec 13;15(12):1329. doi: 10.3390/brainsci15121329 (PMC12730659; doi:10.3390/brainsci15121329)
Supplement: Supplementary file 1 [file brainsci-15-01329-s001.zip › brainsci-3974644-supplementary.pdf]

Supplementary Figures

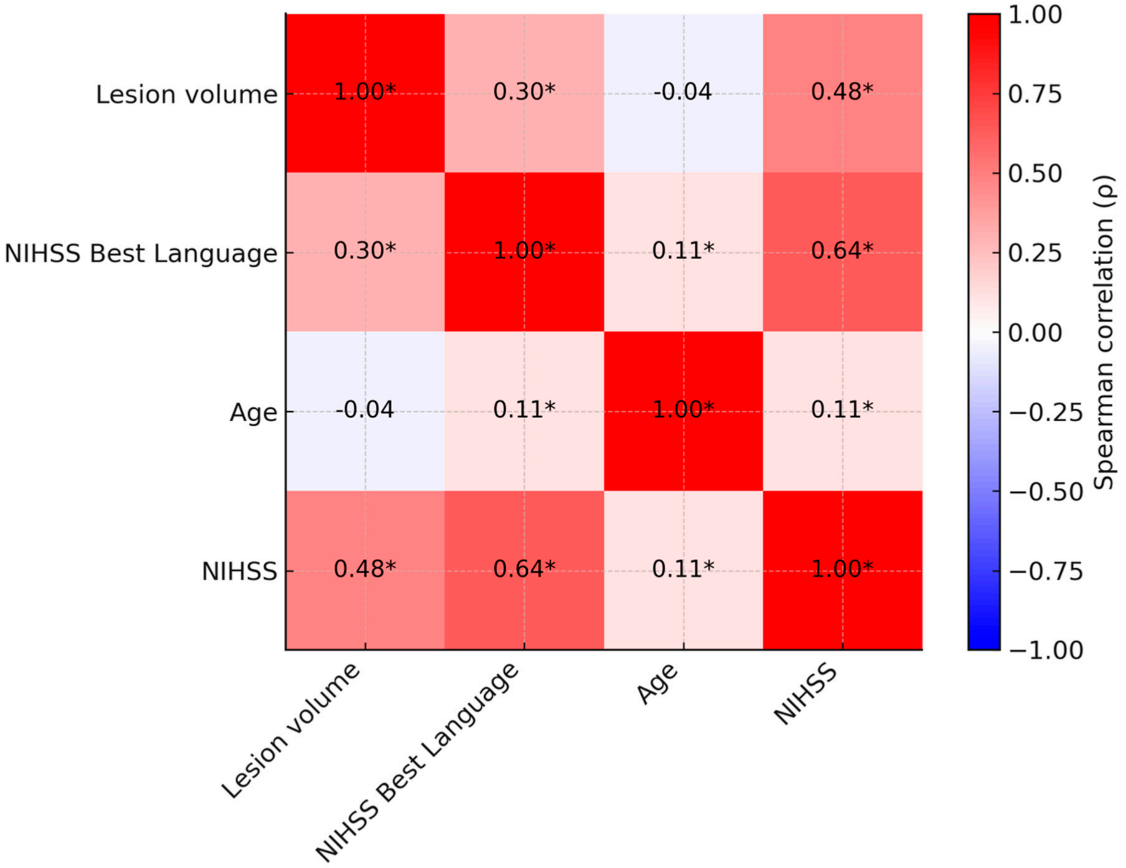

Supplementary Figure S1. Spearman's Correlation Between Lesion Volume, NIHSS Best Language Score, Age, and NIHSS Score. Colorbar represents the p-value of the correlation coefficient. *p < 0.05.

Supplementary Tables

Brain region	JHU ROI #	Z
**Presence of speech deficits (Z [-3.05, 2.91])**		
L External capsule	137	3.22
L Posterior limb of internal capsule	133	3.22
R Posterior insula	183	3.20
R External capsule	138	3.16
L Putamen	79	3.16
L Anterior limb of internal capsule	131	3.06
L Superior fronto-occipital fasciculus	153	2.92
R Insula	72	2.91
**Severity of speech deficits (Z [-3.01, 3.26])**		
L External capsule	137	6.39
L Posterior insula	182	5.64
L Putamen	79	5.52
L Inferior fronto-occipital fasciculus	147	5.43
L Superior corona radiata	119	4.76
L Anterior limb of internal capsule	131	4.76
L Insula	71	4.71
L Caudate nucleus	77	4.53
L Inferior frontal gyrus pars opercularis	11	4.30
L Posterior limb of internal capsule	133	4.29
L Superior fronto-occipital fasciculus	153	4.26
L Retrolenticular part of internal capsule	135	3.92
L Uncinate fasciculus	157	3.73
L Precentral gyrus	25	3.57
L Pole of superior temporal gyrus	37	3.50
L Superior temporal gyrus	35	3.31
R Genu of corpus callosum	124	-3.57
L Precuneus	33	-3.49
R Splenium of corpus callosum	128	-3.47
R Body of corpus callosum	126	-3.45
L Posterior cingulate gyrus	69	-3.40
R Middle occipital gyrus	54	-3.03

Supplementary Table S1. Region-of-Interest Lesion-Symptom Mapping Results for the Full Sample (N = 410). Regional effects that survived N = 5,000 permutation thresholding. Z brackets indicate adjusted statistical thresholds, and higher Z-scores represent greater lesion load associated with presence and greater severity of speech deficits. Abbreviations: JHU = Johns Hopkins University, L = left, R = right, ROI = region-of-interest.

Brain region	JHU ROI #	Z
**Presence of speech deficits (Z [-2.97, 2.64])**		
L External capsule	137	3.77
L Superior corona radiata	119	3.68
L Posterior limb of internal capsule	133	3.68
L Inferior fronto-occipital fasciculus	147	3.51
L Anterior limb of internal capsule	131	3.49
L Putamen	79	3.47
L Superior fronto-occipital fasciculus	153	3.41
L Caudate nucleus	77	3.26
L Retrolenticular part of internal capsule	135	2.95
L Posterior insula	182	2.84
L Fornix / Stria terminalis	143	2.67
**Severity of speech deficits (Z [-2.90, 2.91])**		
L External capsule	137	6.07
L Inferior fronto-occipital fasciculus	147	5.19
L Posterior insula	182	5.14
L Putamen	79	4.90
L Superior corona radiata	119	4.79
L Anterior limb of internal capsule	131	4.29
L Insula	71	4.15
L Caudate nucleus	77	4.10
L Superior fronto-occipital fasciculus	153	3.83
L Inferior frontal gyrus pars opercularis	11	3.78
L Posterior limb of internal capsule	133	3.68
L Retrolenticular part of internal capsule	135	3.68
L Precentral gyrus	25	3.62
L Uncinate fasciculus	157	3.51
L Pole of superior temporal gyrus	37	3.13
L Posterior cingulate gyrus	69	-3.25
L Splenium of corpus callosum	127	-2.91
L Precuneus	33	-3.81

Supplementary Table S2. Region-of-Interest Lesion-Symptom Mapping Results for the Left Hemisphere Sample (N = 225). Regional effects that survived N = 5,000 permutation thresholding. Z brackets indicate adjusted statistical thresholds, and higher Z-scores represent greater lesion load associated with presence and greater severity of speech deficits. Abbreviations: JHU = Johns Hopkins University, L = left, R = right, ROI = region-of-interest.

Brain region	JHU ROI #	Z
**Presence of speech deficits (Z [-2.80, 2.81])**		
R Posterior insula	183	3.70
R External capsule	138	3.63
R Insula	72	3.09
R Retrolenticular part of internal capsule	136	3.02
**Severity of speech deficits (Z [-2.68, 3.18])**		
R External capsule	138	3.59
R Posterior insula	183	3.46
R Middle fronto-orbital gyrus	20	-3.03

Supplementary Table S3. Region-of-Interest Lesion-Symptom Mapping Results for the Right Hemisphere Sample (N = 185). Regional effects that survived N = 5,000 permutation thresholding. Z brackets indicate adjusted statistical thresholds, and higher Z-scores represent greater lesion load associated with presence and greater severity of speech deficits. Abbreviations: JHU = Johns Hopkins University, L = left, R = right, ROI = region-of-interest.

Peak Z	Voxels	Coordinates	Structures
**Presence of speech deficits (Z [-4.41, 4.28])**			
5.1	2,475	37.8*2.1*2.7	R Insula, R External capsule, R Precentral gyrus, R Posterior insula, R Postcentral gyrus, R Retrolenticular part of internal capsule, R Superior temporal gyrus
4.9	33	-30.0*-5.3*13.0	L External capsule, L Insula, L Posterior insula
4.7	178	-19.7*1.3*15.3	L Anterior limb of internal capsule, L Caudate nucleus, L Superior fronto-occipital fasciculus
4.6	45	-28.6*1.3*10.1	L External capsule, L Putamen
4.6	100	-26.4*15.3*-1.0	L External capsule, L Putamen
4.5	34	46.7*-14.9*6.4	R Superior temporal gyrus
**Severity of speech deficits (Z [-4.05, 4.60])**			
6.8	10,875	-32.3*-7.5*7.9	L External capsule, L Posterior insula, L Insula, L Precentral gyrus, L Putamen, L Inferior frontal gyrus pars opercularis, L Postcentral gyrus
5.3	261	-25.6*10.2*22.6	L Superior corona radiata, L Precentral gyrus, L Inferior frontal gyrus pars opercularis
5.1	85	-55.1*12.4*23.4	L Precentral gyrus, L Inferior frontal gyrus pars opercularis
5.0	112	-15.3*-1.6*16.0	L Caudate nucleus, L Anterior limb of internal capsule, L Thalamus
4.9	47	-13.1*13.1*6.4	L Caudate nucleus, L Anterior limb of internal capsule

Supplementary Table S4. Voxelwise Lesion-Symptom Mapping Results for the Full Sample (N = 410). Clusters that survived N = 5,000 permutation thresholding. Z brackets indicate adjusted statistical thresholds, and higher Z-scores represent greater lesion load associated with presence and greater severity of speech deficits. Abbreviations: L = left, R = right.

Peak Z	Voxels	Coordinates	Structures
**Presence of speech deficits (Z [-4.37, 4.01])**			
4.4	67	-19.7*1.3*14.5	L Anterior limb of internal capsule
4.3	53	-26.4*15.3*-1.0	L External capsule, L Putamen
**Severity of speech deficits (Z [-4.08, 4.23])**			
6.4	8,323	-33.0*-5.3*4.9	L External capsule, L Posterior insula, L Insula, L Precentral gyrus, L Putamen, L Postcentral gyrus, L Inferior fronto-occipital fasciculus, L Inferior frontal gyrus pars opercularis
5.0	237	-25.6*10.2*22.6	L Superior corona radiata, L Precentral gyrus, L Inferior frontal gyrus pars opercularis
4.9	43	-50.7*7.2*-3.9	L Pole of superior temporal gyrus, L Precentral gyrus
4.8	45	-55.1*5.0*14.5	L Precentral gyrus
4.6	37	-19.7*2.1*14.5	L Anterior limb of internal capsule
4.6	57	-55.1*12.4*23.4	L Precentral gyrus, L Inferior frontal gyrus pars opercularis
4.5	49	-16.8*-6.0*16.7	L Caudate nucleus, L Thalamus, L Anterior limb of internal capsule

Supplementary Table S5. Voxelwise Lesion-Symptom Mapping Results for the Left Hemisphere Sample (N = 225). Clusters that survived N = 5,000 permutation thresholding. Z brackets indicate adjusted statistical thresholds, and higher Z-scores represent greater lesion load associated with presence and greater severity of speech deficits. Abbreviations: L = left, R = right.

Peak Z	Voxels	Coordinates	Structures
**Presence of speech deficits (Z [-4.16, 4.18])**			
6.3	24,800	37.8*2.1*2.7	R Precentral gyrus, R Supramarginal gyrus, R Insula, R Superior temporal gyrus, R Postcentral gyrus, R Posterior insula, R External capsule
4.9	448	55.5*-40.0*27.8	R Supramarginal gyrus, R Angular gyrus
4.7	59	62.1*-11.2*4.9	R Superior temporal gyrus
4.5	44	49.6*-5.3*41.1	R Precentral gyrus, R Postcentral gyrus
**Severity of speech deficits (Z [-3.75, 4.47])**			
5.5	130	37.8*2.1*2.7	R Insula, R External capsule, R Posterior insula, R Inferior fronto-occipital fasciculus

Supplementary Table S6. Voxelwise Lesion-Symptom Mapping Results for the Right Hemisphere Sample (N = 185). Clusters that survived N = 5,000 permutation thresholding. Z brackets indicate adjusted statistical thresholds, and higher Z-scores represent greater lesion load associated with presence and greater severity of speech deficits. Abbreviations: L = left, R = right.
